# Integrin β Regulates the Hepatopancreas Antiviral Innate Immune System by Affecting the Expression of Antimicrobial Peptides in *Penaeus vannamei*

**DOI:** 10.3390/ijms26178478

**Published:** 2025-08-31

**Authors:** Bingbing Yang, Li Zhang, Kun Luo, Sheng Luan, Jie Kong, Qiang Fu, Jiawang Cao, Baolong Chen, Ping Dai, Xupeng Li, Xianhong Meng

**Affiliations:** 1 State Key Laboratory of Mariculture Biobreeding and Sustainable Goods, Yellow Sea Fisheries Research Institute, Chinese Academy of Fishery Sciences, Qingdao 266071, China; 2 Laboratory for Marine Fisheries Science and Food Production Processes, Qingdao Marine Science and Technology Center, Qingdao 266237, China; 3 College of Fisheries and Life Science, Shanghai Ocean University, Shanghai 201306, China

**Keywords:** *Penaeus vannamei*, Integrin β, white spot syndrome virus, innate immune

## Abstract

*Penaeus vannamei* aquaculture production accounts for the majority of total shrimp aquaculture output, but it has suffered a severe decline in production and economic losses due to WSSV disease. Therefore, elucidating the relationship between the host immune system and pathogens is crucial for shrimp disease prevention and control. Integrins, as receptor-related molecules, have been shown to participate in various physiological functions, including cell migration, organismal development, and the pathogenesis of multiple diseases. However, the regulatory mechanisms of integrin genes in the shrimp immune system remain unclear. This study reports that integrins may regulate the Toll, IMD, and STAT signaling pathways in *P. vannamei* by influencing *Spätzle*, *TLR*, and *Domeless*, thereby affecting the shrimp’s innate immune system against diseases. Additionally, integrins can inhibit viral entry and replication. Through RNA interference (RNAi) experiments, it was found that knocking down *Pv*-Integrin β increases the viral load of white spot syndrome virus (WSSV), making shrimp more susceptible to WSSV and giving rise to increasing mortality. Further research indicates that *Pv*-Integrin β acts as an upstream recognition receptor in the disease resistance immune pathway, influencing other signaling pathway receptors to regulate the innate immune system. Importantly, knocking down *Pv*-Integrin β upregulates the expression of antimicrobial peptides such as *ALF1* and *ALF2*, but reduces the expression of *Crustin1*, *Crustin2* and prophenoloxidase. In conclusion, this study reveals that *Pv*-Integrin β regulates the disease resistance immune signaling pathways by affecting the related receptors.

## 1. Introduction

White spot syndrome virus (WSSV) can infect not only shrimp but also crabs and other crustaceans [1]. The virus first emerged in the region of Asia in the 1990s and subsequently spread to shrimp farming regions worldwide, causing severe mortality rates and substantial economic losses. Despite extensive research over the past few decades, no effective treatment is currently available. WSSV belongs to the genus *Whispovirus* in the family *Nimaviridae*, and is a rod-shaped, double-stranded DNA (dsDNA) virus with a genome size of approximately 300 kbp [2,3]. Although a series of studies have been conducted, the molecular mechanisms by which the virus regulates the host cells remain incompletely understood. Further exploration of the molecular interactions between WSSV and its host may provide potential therapeutic targets for white spot syndrome.

According to research reports, the process of viral infection involves two distinct stages: attachment to the cell surface and entry into the cell [4]. In fact, viral infection of the host cells primarily occurs through the viral envelope proteins, which facilitate intracellular assembly and the budding process [5]. Taking WSSV as an example, multiple envelope protein structures have been identified. However, a recent study revealed that 58 structural proteins have been identified in WSSV, including nucleocapsid proteins and envelope proteins, with the VP series (such as VP 19, VP 24, VP 26, and VP 28) being the most predominant [6,7]. During viral infection, studies have shown that host cell membrane components can act as receptors by binding to viral envelope proteins, thereby promoting viral penetration. In crustaceans, known receptor components involved in viral recognition include Toll receptors, C-type lectins (CTL), scavenger receptors (SR), β-integrins, polymeric immunoglobulin receptors (pIgR), laminin receptors, globular C1q receptors (gC1qR), lipopolysaccharides (LPS), β-1,3-glucan binding proteins (LGBP), chitin binding proteins (CBP), Ras-associated binding proteins (Rab), and Down syndrome cell adhesion molecules (Dscam) [8]. Although the extent of research on these receptor molecules varies, they hold significant potential in pathogen blockade defense and target therapy, with receptor genes undoubtedly being the optimal choice.

Notably, integrins are a class of heterodimeric cell surface receptors composed of α and β subunits, playing a crucial role in mediating cell–matrix and cell–cell interactions, as well as bidirectional signal transduction [9]. Each subunit consists of three main regions: an extracellular domain, a transmembrane domain, and a small cytoplasmic domain [10]. Research has demonstrated that integrins are essential in various physiological processes, including cell migration, development, wound healing, immune system regulation, and the pathogenesis of multiple diseases [11]. Additionally, as cellular receptors, integrins have been shown to facilitate viral internalization into host cells, a function that is particularly critical in the early stages of viral infection [12,13]. It is noteworthy that integrins, as heterodimeric proteins, have been extensively studied in multiple species, including humans, zebrafish, and fruit flies [9]. However, despite an in-depth exploration of integrin functions in these species, the existence of their subunits in crustaceans has not yet been definitively confirmed [14]. This research gap highlights the importance of future studies to explore the functions and potential roles of integrins in crustaceans. In crustaceans, the *Integrin β* gene of *Fenneropenaeus chinensis* is distributed in the hemocytes, heart, gills, gonads, intestines, muscles, and hepatopancreas, with varying expression levels across these tissues. Notably, the *Fc-Integrin β* gene is significantly induced in gill tissues following viral infection [14]. Additionally, in a study on *Penaeus vannamei*, the *Integrin β* gene was successfully cloned from hemocytes. RNA interference (RNAi) experiments revealed that *Integrin β* plays a crucial role in regulating shrimp immune responses, including the modulation of the prophenoloxidase system, phagocytosis, and the antioxidant system [15]. However, these studies have yet to elucidate the specific disease resistance pathways through which the *Integrin β* gene participates in the innate immune response of shrimp.

Invertebrates lack the adaptive immune response system found in vertebrates and are, therefore, believed to rely entirely on their innate immune system to combat viral pathogens. When pattern recognition receptors (PRRs) are activated by viral components, the innate antiviral response is rapidly triggered. This response encompasses both humoral and cellular immunity. In invertebrates, the humoral immune response primarily involves the prophenoloxidase (proPO) system, the coagulation cascade, and the production of various antimicrobial peptides. Meanwhile, the cellular immune response includes mechanisms such as apoptosis and phagocytosis. In aquatic crustaceans, the innate immune system plays a crucial role as an essential defense mechanism against pathogens [16].

Through this study, it was found that the Integrin β molecule of *P. vannamei* can respond to an infection of WSSV. As an important economic aquaculture species, *P. vannamei* has long been affected by pathogens. By using RNAi technology to knock down the *Integrin β* gene, we aim to validate its role in the molecular regulation mechanism of the shrimp’s innate immune response against pathogens. The *Integrin β* gene plays a role in influencing the Toll, IMD, and STAT pathways, as shown during this study, providing new insights into resistance and immune mechanisms between pathogens and hosts.

## 2. Results

### 2.1. Pv-Integrin β Gene Sequence Information

**Figure 1:**
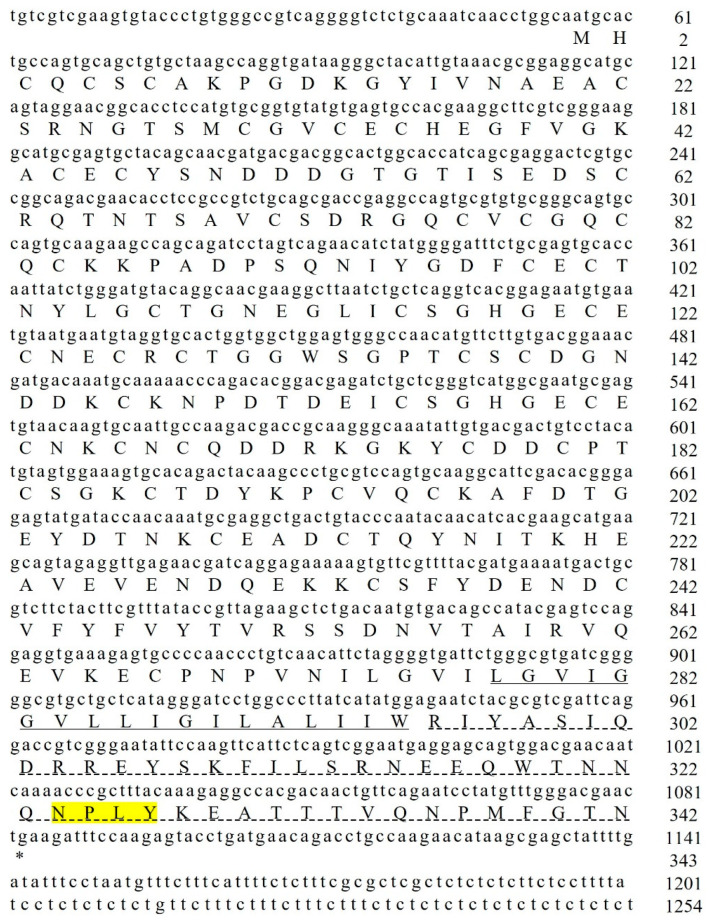
The cDNA sequence of *Pv-Integrin β* is shown in the figure, with lowercase letters representing the gene sequence and uppercase letters representing the amino acid sequence. It contains a conserved “NPxY” motif (yellow area), a transmembrane domain, and a cytoplasmic domain.

In our previous study, we identified a gene sequence (mRNA sequence LOC113805717, amino acid sequence XM_027356770.1) from the transcriptome sequencing data of *P. vannamei* infected with WSSV. Subsequently, we amplified a 1254 bp sequence via PCR and sent it to Sangon Biotech (Shanghai) for sequencing. Based on the sequencing results, we performed basic bioinformatics analysis and aligned the sequence using the BLAST 2.17.0 software (http://blast.ncbi.nlm.nih.gov/Blast.cgi, accessed on 20 August 2024). According to the sequence’s characteristics, we designated it as the *Pv-Integrin β* gene. This gene contains an open reading frame (ORF) encoding 342 amino acids, with a predicted theoretical molecular weight of 37.6 kDa. Further analysis revealed that this gene contains a conserved “NPxY” motif (marked as a yellow area) and a transmembrane domain and cytoplasmic domain, indicated with solid underlines and dashed underlines, respectively (Figure 1).

### 2.2. Multiple Sequence Alignment and Phylogenetic Tree

**Figure 2:**
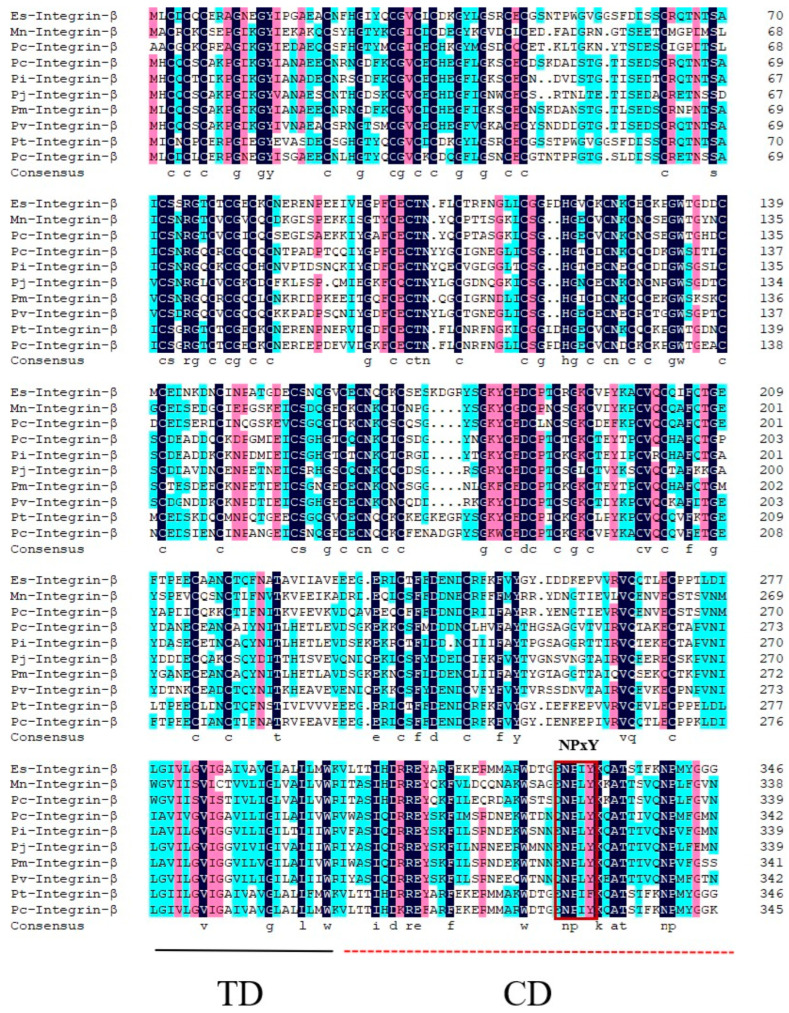
The multiple sequence alignment analysis of the *Pv*-Integrin β molecule. The numbers on the right indicate the amino acid positions. The analysis reveals that this molecule contains a transmembrane domain and a cytoplasmic domain, with a conserved “NPxY” motif highlighted in a red box. In the alignment results, the different colors represent the conservation levels of amino acids: black indicates complete conservation, red indicates high conservation, and blue indicates moderate conservation.

Using BLAST, we aligned the amino acid sequence of *Pv*-Integrin β and found that it shares high similarity (67.17%) with Integrin β sequences from several species, including *Penaeus chinensis*-Integrin β (XP_047479545.1), *Penaeus monodon*-Integrin β (XP_037801846.1), *Penaeus japonicus*-Integrin β (XP_042886327.1), *Procambarus clarkii*-Integrin β (XP_045617424.1), *Eriocheir sinensis*-Integrin β (XP_050729686.1), *Macrobrachium nipponense*-Integrin β (XP_064077230.1), *Palaemon carinicauda*-Integrin β (XP_068210289.1), *Penaeus indicus*-Integrin β (XP_063601209.1), and *Portunus trituberculatus*-Integrin β (XP_045106617.1). Further analysis revealed that *Pv*-Integrin β contains a conserved “NPxY” motif, as well as a transmembrane domain (TD) and a cytoplasmic domain (CD), which are also present in Integrin β sequences from other species (Figure 2).

**Figure 3:**
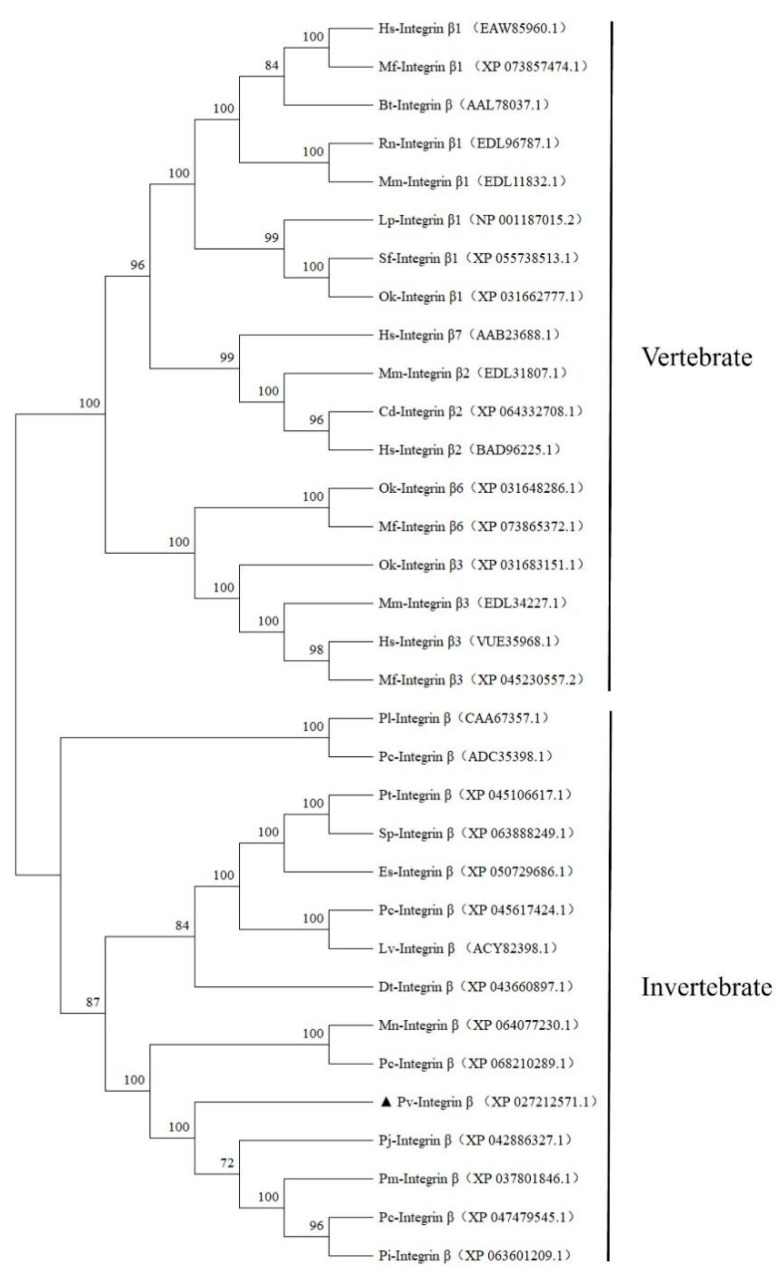
The phylogenetic tree of *Pv*-Integrin β with Integrin β molecules from both vertebrates and invertebrates. The Δ symbol represents the Integrin β molecule of *P. vannamei*. The neighbor-joining tree was constructed using MEGA 11.0 software, and the results were validated with 1000 bootstrap replicates to ensure reliability.

Based on the amino acid sequence alignment, we constructed a phylogenetic tree using MEGA11.0 software. Since *P. vannamei* is an invertebrate, we selected both invertebrate and vertebrate species for comparison. The invertebrate species included *P. chinensis*-Integrin β (XP_047479545.1), *P. japonicus*-Integrin β (XP_042886327.1), *P. clarkii*-Integrin β (XP_045617424.1), *E. sinensis*-Integrin β (XP_050729686.1), *M. nipponense*-Integrin β (XP_064077230.1), *P. carinicauda*-Integrin β (XP_068210289.1), *P. indicus*-Integrin β (XP_063601209.1), *P. trituberculatus*-Integrin β (XP_045106617.1), *Scylla paramamosain*-Integrin β (XP_063888249.1), *Drosophila teissieri*-Integrin β (XP_043660897.1), *L*. *vannamei*-Integrin β (ACY82398.1), *P. chinensis*-Integrin β (ADC35398.1), *P. leniusculus*-Integrin β (CAA67357.1), and *P. monodon*-Integrin β (XP_037801846.1). The vertebrate species included *Rattus norvegicus*-Integrin β1 (EDL96787.1), *Bos taurus*-Integrin β1 (AAL78037.1), *Camelus dromedarius*-Integrin β2 (XP_064332708.1), *Salvelinus fontinalis*-Integrin β1 (XP_055738513.1), *Ictalurus punctatus*-Integrin β1 (NP_001187015.2), *Homo sapiens*-Integrin β7 (AAB23688.1), *Homo sapiens*-Integrin β3 (VUE35968.1), *Homo sapiens*-Integrin β1 (EAW85960.1), *Homo sapiens*-Integrin β2 (BAD96225.1), *Mus musculus*-Integrin β1 (EDL11832.1), *Mus musculus*-Integrin β3 (EDL34227.1), *Mus musculus*-Integrin β2 (EDL31807.1), *Oncorhynchus kisutch*-Integrin β1 (XP_031662777.1), *Oncorhynchus kisutch*-Integrin β6 (XP_031648286.1), *Oncorhynchus kisutch*-Integrin β3 (XP_031683151.1), *Macaca fascicularis*-Integrin β1 (XP_073857474.1), *Macaca fascicularis*-Integrin β6 (XP_073865372.1), and *Macaca fascicularis*-Integrin β3 (XP_045230557.2). The phylogenetic tree revealed that Integrin β sequences from the vertebrates and invertebrates formed two distinct clades, with vertebrates grouped into one branch and invertebrates into another. Within the invertebrate clade, *Pv*-Integrin β clustered closely with *M. nipponense*-Integrin β (XP_064077230.1), *P. japonicus*-Integrin β (XP_042886327.1), and *P. carinicauda*-Integrin β (XP_068210289.1)*,* indicating a close evolutionary relationship. The amino acid sequences of *L. vannamei*-Integrin β (ACY82398.1) and *Pv*-Integrin β form two independent branches in the phylogenetic tree, despite both sequences belonging to the invertebrate clade. However, *L. vannamei*-Integrin β (ACY82398.1) and *P. clarkii*-Integrin β (XP_045617424.1) are on the same branch, indicating a closer phylogenetic relationship. In contrast, *Pv*-Integrin β showed a lower level of evolution with *H. sapiens*-Integrin β1 (EAW85960.1) and *M. fascicularis*-Integrin β1 (XP_073857474.1) (Figure 3).

### 2.3. Analysis of Pv-Integrin β Expression Patterns Following WSSV Infection

**Figure 4:**
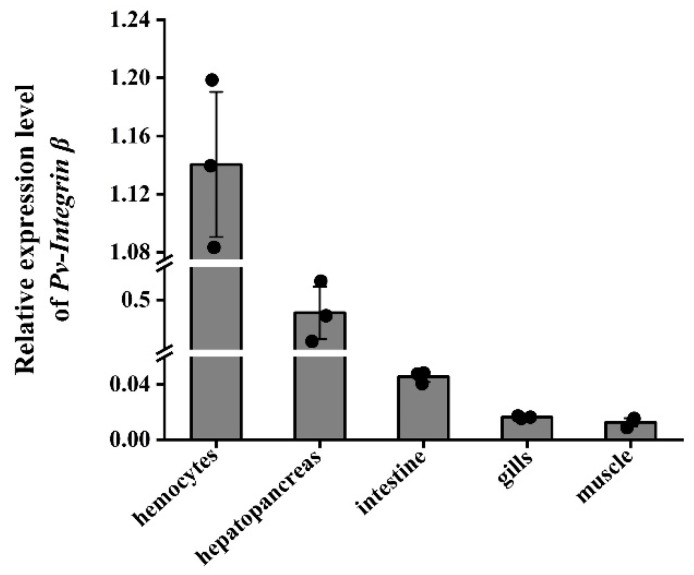
The tissue distribution of the *Pv-Integrin β* gene in normal *P. vannamei* was detected using qRT-PCR technology, using 18 S rRNA as an internal control. Three parallel experiments were performed to ensure the reliability of the results (including three biological replicates). A histogram was used to display the results of the statistical analysis. The data were analyzed using the 2^−ΔΔCT^ method.

We first analyzed the relative expression levels of the *Pv-Integrin β* gene in various tissues of healthy *P. vannamei* using qRT-PCR. The results showed that among the five tissues examined (hemocytes, hepatopancreas, the intestines, gills, and muscle), the *Pv-Integrin β* gene exhibited the highest expression level in hemocytes and the lowest in muscle tissue (Figure 4).

**Figure 5:**
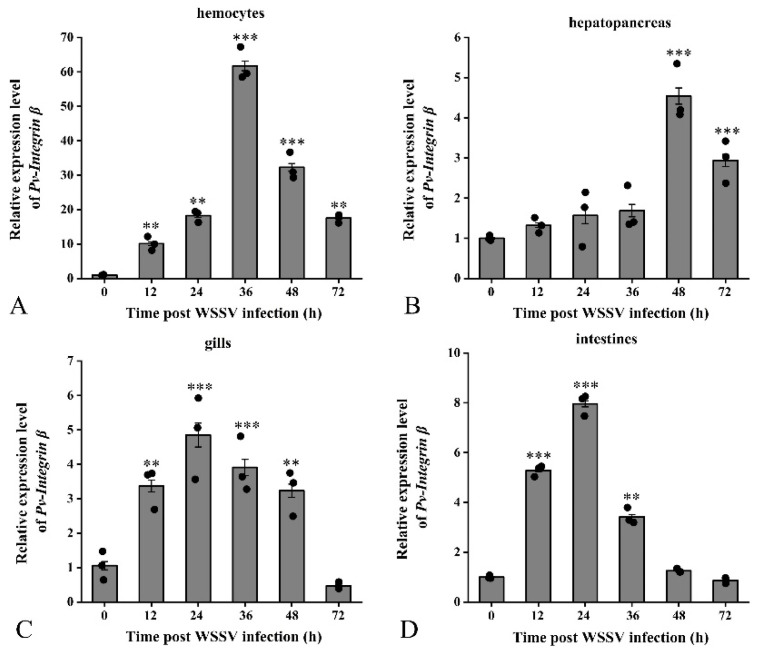
After infection with WSSV, the expression pattern of the *Pv-Integrin β* gene is as follows: (**A**) shows the expression level of the *Pv-Integrin β* gene in hemocytes, (**B**) shows the relative expression level in hepatopancreas tissue, (**C**) shows the relative expression level in gill tissue, and (**D**) shows the relative expression level in intestine tissue. The experiment was independently repeated three times and included three biological replicates. The data were analyzed using the 2^−ΔΔCT^ method, and the results were compared using an unpaired two-tailed *t*-test. Statistical analysis of the data was conducted using an ANOVA (IBM SPSS Statistics 22, accessed on 27 September 2024). The results are expressed as mean ± standard deviation. Significant differences compared to the control group are indicated by asterisks; ** *p* < 0.01 indicates an extremely significant difference, and *** *p* < 0.001 indicates an exceptionally significant difference.

Furthermore, we examined the expression changes of the *Pv-Integrin β* gene in hemocytes, hepatopancreas, gills, and the intestine after *P. vannamei* was infected with WSSV. The results demonstrated that WSSV infection significantly upregulates the expression of the *Pv-Integrin β* gene in these four tissues. Specifically, in hemocytes, the relative expression level of *Pv-Integrin β* peaked at 36 hpi, increasing up to 60-fold (Figure 5A). In the hepatopancreas, the expression level rose to 4.5-fold at 48 hpi (Figure 5B). In the gills, the expression level increased to 4.8-fold at 24 hpi (Figure 5C), while in the intestines, the gene expression level reached 7.8-fold at 24 hpi (Figure 5D).

These experimental findings reveal that WSSV infection induces the significant upregulation of the *Pv-Integrin β* gene in multiple tissues of *P. vannamei*, particularly in hemocytes. This suggests that the *Pv-Integrin β* gene may play a role in the antiviral immune response of *P. vannamei*.

### 2.4. Analysis of dsRNA Interference Efficiency and Survival Rate of P. vannamei

**Figure 6:**
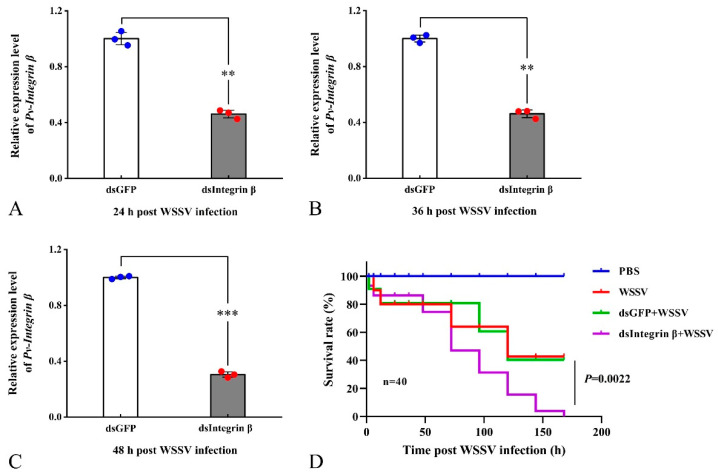
The effective knockdown of the target gene and the increased mortality of shrimp after an injection of dsIntegrin β RNA: (**A**–**C**) demonstrates the interference effect validation at 24, 36, and 48 h, while (**D**) shows a statistical analysis of shrimp survival rates. Compared to the control group (dsGFP + WSSV), the survival rate of the dsIntegrin β + WSSV group showed a significant decreasing trend. Statistical analysis of the data was conducted using an ANOVA. This survival rate-related statistical experiment was independently repeated three times. Significant differences compared to the control group are indicated by asterisks (** *p* < 0.01, *** *p* < 0.001).

To verify the interference effect of dsRNA injection, we measured the relative expression level of the *Pv-Integrin β* gene after dsRNA injection using qRT-PCR. The experimental results showed that, compared to the control group (dsGFP + WSSV), the target gene was effectively knocked down at different time points after the injection of dsIntegrin β + WSSV. Specifically, the interference efficiency of *Pv-Integrin β* remained at 54%, 58%, and 70% at 24, 36, and 48 hpi, respectively (Figure 6A–C). Subsequently, we statistically analyzed the survival rates of *P. vannamei* in four groups: PBS, WSSV, dsGFP + WSSV, and dsIntegrin β + WSSV. The results revealed that, compared to the control group (dsGFP + WSSV), the survival rate of shrimp in the dsIntegrin β + WSSV group showed a significant downward trend (Figure 6D).

### 2.5. Knockdown of the Pv-Integrin β Gene Promotes WSSV replication

**Figure 7:**
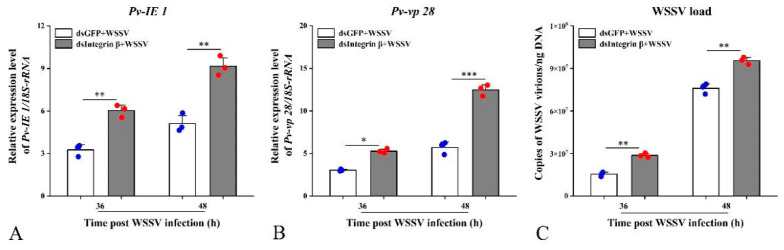
Graphs demonstrating that the knockdown of the target gene promotes WSSV replication. The injection of dsIntegrin β RNA, combined with WSSV, was used to validate its antiviral activity in vivo. The qRT-PCR results showed that compared to the control group (dsGFP + WSSV), knockdown of the target gene significantly upregulated the expression levels of *IE 1* and *vp 28* (**A**,**B**). The detection of WSSV viral load revealed that the WSSV load in the dsIntegrin β + WSSV group was significantly increased at 36 and 48 h (**C**). All experiments were independently repeated three times. The data were analyzed using the 2^−ΔΔCT^ method, and the results were compared using an unpaired two-tailed *t*-test. Statistical analysis of the data was conducted using an ANOVA. Significant differences compared to the control group are indicated by asterisks; * *p* < 0.05 indicates a significant difference, ** *p* < 0.01 indicates an extremely significant difference, and *** *p* < 0.001 indicates an exceptionally significant difference.

To further investigate the role of the *Pv-Integrin β* gene in antiviral immunity in shrimp, the study employed a knockdown approach targeting *Pv-Integrin β* to assess its impact on WSSV replication. Furthermore, the expression levels of the key WSSV proteins *IE 1* and *vp 28* were analyzed, revealing that the knockdown of *Pv-Integrin β* led to a significant upregulation of these viral proteins (Figure 7A,B). These findings suggest that *Pv-Integrin β* may play a critical role in shrimp antiviral immunity against WSSV. In order to more accurately detect the viral load, a probe-based method was used to quantify the WSSV copy numbers. Compared to the dsGFP + WSSV control group, the knockdown of *Pv-Integrin β* significantly enhanced WSSV replication in infected shrimp (the WSSV load significantly increased at 36 and 48 h; see Figure 7C).

### 2.6. Role of Pv-Integrin β in the Regulation of Innate Immune Pathways

**Figure 8:**
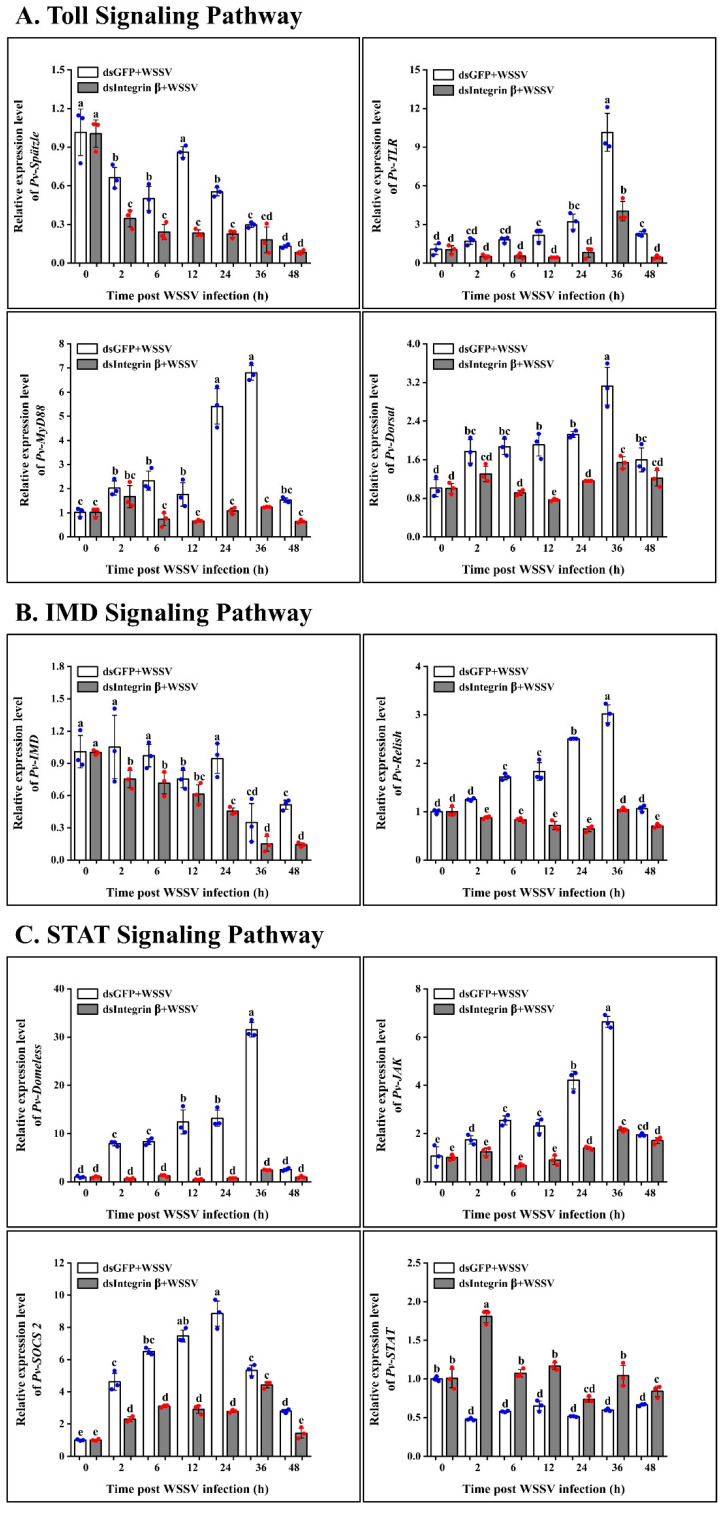

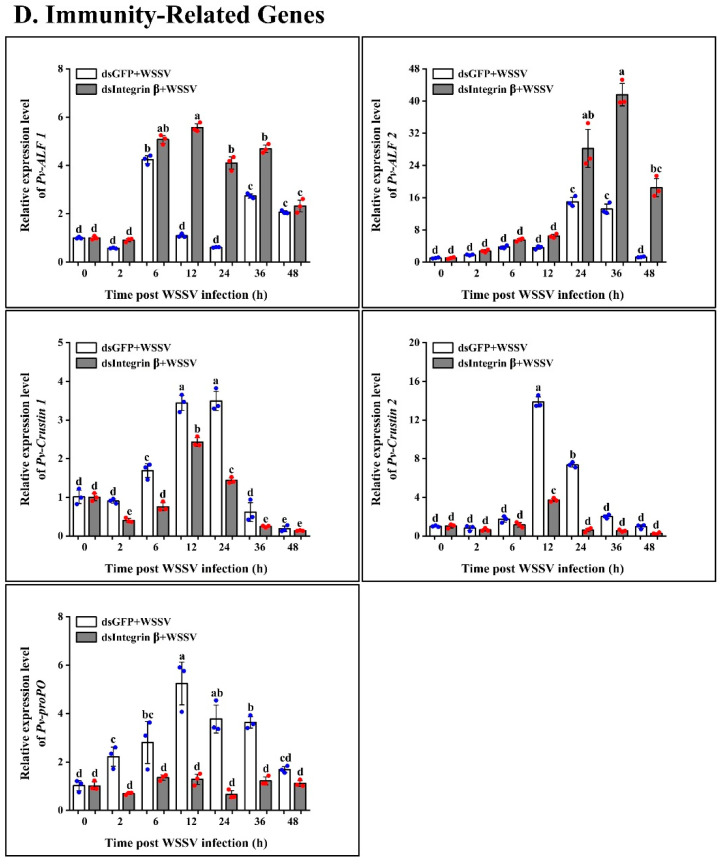
*Integrin β* regulates immune genes through the Toll, IMD, and STAT signaling pathways: (**A**) shows the Toll signaling pathway; (**B**) illustrates the IMD signaling pathway; (**C**) demonstrates the STAT signaling pathway; (**D**) presents the immune-related genes. The experiment was independently repeated three times and included three biological replicates. The data were analyzed using the 2^−ΔΔCT^ method, and the results were compared using an unpaired two-tailed *t*-test. Statistical analysis of the data was conducted using an ANOVA. Compared with the control group, identical letters indicate no significant difference (*p* > 0.05), while different letters signify statistically significant differences between groups (*p* < 0.05).

*Pv-Integrin β*, as an important membrane receptor gene, plays a crucial role in anti-disease innate immunity. To further elucidate its regulatory mechanism, we examined the expression levels of *Toll*, *IMD*, the *STAT* signaling pathways, and related effector molecules. The study found that in the Toll signaling pathway, knocking down the *Pv-Integrin β* gene resulted in a significant decrease in the expression levels of *Spätzle*, *TLR*, *MyD88*, and *Dorsal* (Figure 8A). Similarly, in the IMD signaling pathway, the expression levels of *IMD* and *Relish* also significantly decreased (Figure 8B). However, in the STAT signaling pathway, the expression levels of *Domeless*, *JAK*, and *SOCS2* significantly decreased, while *STAT* expression levels significantly increased (Figure 8C). In the family of antimicrobial peptides (AMP), antilipopolysaccharide factors *ALF1* and *ALF2* are significantly upregulated, while *Crustin1* and *Crustin2* are significantly downregulated. Additionally, *proPO* also shows a significant downward trend (Figure 8D). These results suggest that *Pv-Integrin β* may regulate the expression of antiviral pathway genes in shrimp by modulating the Toll, IMD, and STAT signaling pathways, thereby playing a crucial role in innate immune responses.

## 3. Discussion

Integrins are adhesion molecules that are located on the cell surface, playing a significant role in an organism’s growth, development, and immune response. Additionally, they regulate various cellular processes, including proliferation, differentiation, adhesion, migration, and apoptosis [17]. In our study, *Pv-Integrin β* was identified from the transcriptome data of *P. vannamei* infected with WSSV. Subsequent PCR amplification and sequencing confirmed the ORF of *Pv-Integrin β*. Bioinformatics analysis revealed that *Pv-Integrin β* exhibits evolutionary conservation, particularly the “NPxY” motif, which is consistent across species. Most Integrin β subunits contain at least one conserved NPX [Y/F] motif in their cytoplasmic domains, which serves as a typical phosphotyrosine binding site. This motif is implicated in intracellular signaling and the activation of integrins, facilitating communication between the cell and its environment [18]. Although the *Pv*-Integrin β amino acid sequence used in this study consists of 342 amino acids, this is not the full-length integrin sequence. This may be due to incomplete annotations in the database. However, upon analysis, we found that this sequence covers the key functional domains of the protein, including the transmembrane domain (TD), the cytoplasmic domain (CD), and the “NPxY” motif. Although we attempted to obtain the full-length sequence using 5’-RACE, this was unsuccessful. Nevertheless, this sequence remains of significant research value for elucidating the disease resistance mechanisms in shrimp. However, the sequence analysis may have certain limitations. It is hoped that through further research, the complete integrin beta sequence can be obtained for use in functional comparative studies. Further analysis using qRT-PCR revealed the relative expression levels of the *Pv-Integrin β* gene in various tissues from *P. vannamei*. The results showed that *Pv-Integrin β* exhibited the highest relative expression levels in hemocytes, while the lowest expression levels were observed in muscle tissue. These findings are consistent with previous studies on integrin gene expression in *F. chinensis*, where *Fc-Integrin* was shown to be expressed in various tissues, including the hemocytes, heart, gills, gonads, intestines, muscle, and hepatopancreas, with distinct expression levels across tissues [14].

In the infection experiments, it was observed that WSSV infection induced the upregulation of *Pv-Integrin β* gene expression in the hemocytes, hepatopancreas, gills, and intestine. Interestingly, the upregulation of integrins under WSSV stimulation can activate integrin-associated signaling pathways, thereby promoting the spread of WSSV in the host [12]. In another study on shrimp, it was found that the expression levels of *Integrin α5* and *FAK* mRNA were also significantly upregulated following WSSV infection. By analyzing the phenotypic changes in shrimp following the knockdown of the integrin α5 subunit and β subunit, we found that the magnitude of phenotypic changes induced by α5 knockdown differs from that of β subunit knockdown. Specifically, α5 knockdown has a more significant impact on cell migration and adhesion, while β subunit knockdown primarily affects shrimp survival and pathogen proliferation. These differences may be attributed to the distinct roles of the α5 and β subunits in integrin function [19].

To further investigate the functional roles of integrin genes, we conducted RNAi knockdown experiments targeting the *Pv-Integrin β* gene. To validate the interference efficiency, we confirmed that *Pv-Integrin β* was effectively downregulated at different time points after dsRNA injection. Following the knockdown of *Pv-Integrin β*, a significant decrease in the survival rate of shrimp was observed. Additionally, the knockdown of *Pv-Integrin β* resulted in a significant increase in WSSV infection efficiency and replication capacity. These findings are consistent with a previous study in whiteleg shrimp, where the knockdown of *Pv-Integrin α5* also promoted WSSV replication and reduced shrimp survival [19]. Although both studies focused on *Integrin* genes, they targeted different subtypes. Sequence comparison revealed that these genes are distinct, with the integrins consisting of α and β subunits. However, they may share functional similarities in their roles as receptor genes, potentially mediating the related disease resistance signaling pathways.

The involvement of integrins in classical immune pathways, such as the Toll pathway, IMD pathway, and STAT pathway, remains unclear. To explore the role of *Pv-Integrin β* in disease resistance pathways, we analyzed the hepatopancreas tissue after knockdown of the target gene. In the Toll pathway, qRT-PCR analysis revealed the significant downregulation of *Spätzle*, *TLR*, *MyD88*, and *Dorsal*. The Toll pathway is known to play a critical role in antiviral immunity. Similarly, the IMD pathway also contributes to these defense mechanisms. Our qRT-PCR results showed that the knockdown of *Pv-Integrin β* led to the significant downregulation of *IMD* and *Relish*. However, in the antiviral STAT pathway, knockdown of *Pv-Integrin β* resulted in the significant downregulation of *Domeless*, *JAK*, and SOCS2, while *STAT* exhibited an upregulated expression trend. These findings suggest that *Pv-Integrin β* may influence multiple immune pathways, potentially modulating the shrimp’s antiviral response. The signaling pathways mediated by integrins primarily include the Jak/STAT, MAPK, and PI3K/AKT pathways [20]. However, in invertebrates, reports on the signaling pathways mediated by integrins are scarce, and research into their functional mechanisms is limited.

In mammals, Integrin αvβ3 has been identified as a crucial immune recognition receptor. It can act independently or synergize with Toll-like receptor 2 (TLR2) to recognize various pathogens, including bacteria and viruses, thereby triggering immune defense mechanisms [21]. Both human and mouse models have shown that Integrin αvβ3 plays a key role in combating invading pathogens. Recent studies have highlighted a significant synergistic effect between integrin αvβ3 and TLR2 in mediating immune responses to bacterial lipopeptides, lipopolysaccharides, and viruses. Specifically, in cells co-expressing *Integrin αvβ3* and *TLR2*, the expression levels of interferon alpha and interferon beta are significantly upregulated, along with the enhanced expression of specific cytokines, such as interleukin-2 and interleukin-10. Additionally, nuclear factor κB is activated in response to herpes simplex virus infection or LPS stimulation. Notably, when the integrin β3 subunit is silenced in TLR2-positive cells using gene-silencing techniques, the production of these cytokines and NF-κB activation are significantly reduced [22]. This finding aligns with the conclusions of previous shrimp disease resistance studies, where the inhibition of *Pv-Integrin β* expression led to the significant downregulation of *Dorsal* and *Relish*. In shrimp and other crustaceans, Dorsal, a member of the NF-κB family, plays a role in regulating immune responses. NF-κB, comprising a family of evolutionarily conserved transcription factors, is central to various biological processes [23]. Relish, as a member of the NF-κB family of proteins, plays a crucial role in the immune response. Upon stimulation by pathogens, Relish is cleaved into two fragments within the cytoplasm: the N-terminal fragment containing the Rel homology domain (RHD) and the C-terminal fragment containing the Ankyrin repeat (ANK). The N-terminal fragment, which includes the RHD, is then transported into the nucleus [24]. Here, it binds to the corresponding κB sites in the promoters/enhancers of antimicrobial peptides and other target genes. This binding regulates the transcription of immune-related genes, enabling the organism to mount an effective immune response against the invading pathogens. In crustaceans, studies have shown that *Relish* is involved in immune responses against bacteria, fungi, and WSSV [25,26,27]. Previous studies have reported that the expression of the *Relish* gene is stimulated by *Vibrio harveyi*, WSSV, and yellow head virus [28]. In another study of hemocytes in *Fenneropenaeus chinensis*, *Relish* expression was significantly altered under bacterial and WSSV stimulation [29]. This indicates that Relish participates in the immune response of the organism during bacterial or viral infections.

While there is no direct evidence indicating that integrins regulate the IMD pathway, their involvement in cell adhesion and signal transduction may influence the activation and regulation of the IMD pathway. In shrimp, integrin signaling impacts NF-κB activity, thereby indirectly affecting the IMD pathway’s signaling output. The IMD pathway typically interacts with other immune pathways to regulate immune responses. For instance, the Toll and IMD pathways can synergize to activate innate immune responses [30]. Integrins may influence these interactions, indirectly affecting IMD pathway functionality. Indeed, when *Pv-Integrin β* is interfered with, it inhibits *JAK* expression, while *STAT* expression is upregulated, potentially reflecting regulatory mechanisms within the shrimp. As shown in our experimental results, the knockdown of *Integrin β* affects the *Toll*, *IMD*, and *STAT* transcription factors, regulates the AMPs, and participates in the anti-disease immunity of shrimp. We observed distinct expression profiles for the *ALF* and *Crustin* genes. This differential regulation may be attributed to differences in their upstream signaling pathways and transcriptional regulation. In our study, knocking down *Integrin β* led to significant downregulation trends in certain pathways, such as *Spätzle*, *TLR*, *MyD88*, *IMD*, *Domeless*, *JAK* and *SOCS2*. However, transcription factors such as *Dorsal* and *Relish* were significantly downregulated, while *STAT* showed an upregulation trend. This suggests that the regulation of *ALF* and *Crustin* genes may involve distinct transcriptional mechanisms, with *Dorsal* and *Relish* potentially regulating *Crustin* genes, and *STAT* influencing *ALF* gene expression. Currently, the main research into the pathways regulating antimicrobial peptide families focuses on Toll, IMD, and STAT. The TLR, IMD, and JNK pathways recognize pathogen molecules and activate transcription factors to regulate the expression of antimicrobial peptides. ALFs and Crustins exhibit significant differences in structure, function, and target points; ALFs primarily target Gram-negative bacteria and viruses, while Crustins demonstrate broader antimicrobial and antiviral activities. A study suggests that knocking down integrins may suppress *JAK* expression, but the upregulation of STAT could indicate the viral hijacking of the STAT pathway by WSSV to facilitate its replication. *Lv-SOCS 2*, a negative regulator of the JAK/STAT pathway, has been shown to enhance shrimp resistance to WSSV when knocked out [31]. Simultaneously, it affects the expression levels of *proPO* in the white shrimp *P. vannamei*. Research has confirmed that *Pv-Integrin β* is involved in the activation of proPO and the regulation of the shrimp immune system [15].

In this study, knocking down the *Integrin β* gene in *P. vannamei* resulted in decreased survival rates and increased WSSV copy numbers, primarily due to the attenuation of multiple functions of Integrin β in viral entry and immune regulation. The absence of Integrin β facilitated viral infection and replication in host cells, concomitantly inhibiting the activation of antiviral immune pathways, ultimately leading to increased mortality in shrimp. This study provides crucial insights into the role of Integrin β in antiviral immunity in shrimp and offers new perspectives for developing strategies to combat WSSV.

## 4. Materials and Methods

### 4.1. Experimental Samples Collection

The *P. vannamei* used in the experiment (about 3.6 ± 0.5 g each) were all sourced from BLUP Aquabreed Co., Ltd. (Weifang, China). To ensure the accuracy of the infection experiment, SPF-grade shrimp were selected from the stock of BLUP Aquabreed Co., Ltd. During the experiment, the shrimp were fed twice daily and cultured in sterile seawater for 7 days (salinity 29‰; temperature 25 ± 1 °C). The specific methods were as follows: for the infection experiment, 15 μL of WSSV (4.5 × 10^6^ copies) was injected into the third abdominal segment of each shrimp. Subsequently, the hemolymph, hepatopancreas, gills, and intestines were collected at 12, 24, 36, 48, and 72 h post-infection (hpi). The samples were centrifuged at 4 °C and 3000 rpm for 10 min, and then the hemolymph was separated from the hemocytes. The sampled tissues were stored at ultra-low temperatures for subsequent total RNA extraction experiments.

### 4.2. Total RNA Extraction and cDNA Synthesis

In this experiment, total RNA extraction was performed using the RNA extraction kit provided by Vazyme Biotech Co., Ltd. (Nanjing, China), strictly following the manufacturer’s instructions. For hemocyte tissue, a glass homogenizer was used for homogenization, while other tissues were homogenized mechanically. The extracted total RNA was verified by 1.5% agarose gel electrophoresis, and its concentration was measured. Subsequently, cDNA synthesis was carried out using the First-strand cDNA Synthesis Kit from Vazyme Biotech Co., Ltd. (Nanjing, China).

### 4.3. Gene Cloning and Bioinformatic Analysis

The amplification primers for the *Pv-Integrin β* gene (LOC113805717) were designed using online primer design software (https://primer3.ut.ee/, accessed on 15 August 2024), based on the sequence retrieved from the National Center for Biotechnology Information database. The primer sequences are listed in Table 1. The primers were synthesized by Sangon Biotech Co., Ltd. (Shanghai, China). The ORF sequence of the *Pv-Integrin β* gene was amplified using Quick Taq HS DyeMix (TOYOBO, Shanghai, China), with cDNA from the hepatopancreas of *P. vannamei* as the template. The PCR reaction was performed in a 50 μL system containing 2 μL of cDNA solution (0.1 mg/μL), 25 μL of 2xQuick Taq HS DyeMix (TOYOBO, Shanghai, China), and 1 μL of each primer F/R (10 μM), along with 21 μL of nuclease-free distilled water. The PCR program consisted of initial denaturation at 95 °C for 3 min, followed by 35 cycles of denaturation at 95 °C for 3 s, annealing at 56 °C for 45 s, and extension at 72 °C for 50 s, with a final extension at 72 °C for 5 min. The PCR products were analyzed by 1.0% agarose gel electrophoresis and sent to Sangon Biotech Co., Ltd. (Shanghai, China) for Sanger sequencing. The obtained ORF sequence of the *Pv-Integrin β* gene was translated into an amino acid sequence and used for protein prediction, using the ExPASy online tool (http://www.expasy.org, accessed on 18 August 2024). Subsequently, multiple sequence alignment analysis was performed using the BLAST 2.17.0 online tool (http://blast.ncbi.nlm.nih.gov/Blast.cgi, accessed on 20 August 2024). Finally, a phylogenetic tree was constructed using the DNAMAN 6.0 software (https://www.lynnon.com/, accessed on 10 September 2024).

### 4.4. Tissue Distribution and Expression Pattern Analysis of the Pv-Integrin β Gene

To analyze the relative expression levels of *Pv-Integrin β* in the various tissues of the shrimp, hemocytes, hepatopancreas, gills, intestines, and muscle tissues from healthy *P. vannamei* were selected for an analysis of *Integrin β* expression in these five tissues. In the pathogen infection experiment, 60 shrimp were divided into two groups. Among them, 15 shrimp from the uninfected group were used as the control before infection, and the remaining 45 shrimp would be immunologically infected through injection. The expression levels of the hemocytes, hepatopancreas, gills, intestines, and muscle tissues of the infected shrimp were analyzed. The spatial and temporal expression patterns of *Pv-Integrin β* in normal tissues and after WSSV infection were analyzed using quantitative real-time PCR (qRT-PCR, Applied Biosystems, Foster City, CA, USA) with the SYBR Green Real-time PCR Master Mix Kit (TOYOBO, Shanghai, China). The 18 S ribosomal RNA (18 S rRNA) was used as an internal reference control. The quantitative primers for *Pv-Integrin β*-qRT-F/R and 18 S-F/R are listed in Table 1. The total reaction volume for RT-PCR was 20 μL, containing 10 μL of SYBR PCR mix (TOYOBO, Shanghai, China), 0.8 μL of each primer (10 μM), 2 μL of cDNA solution (0.1 mg/μL), and 6.4 μL of nuclease-free distilled water. The PCR program consisted of initial denaturation at 95 °C for 60 s, followed by 40 cycles of denaturation at 60 °C for 15 s, annealing at 60 °C for 15 s, and extension at 72 °C for 45 s. The data were analyzed using the 2^−ΔΔCT^ method, and the results were compared using an unpaired two-tailed *t*-test. The experiment was independently repeated three times, including three biological replicates. A value of *p* < 0.05 indicates a significant difference, *p* < 0.01 indicates an extremely significant difference, and *p* < 0.001 indicates an exceptionally significant difference.

### 4.5. Double-Stranded RNA Preparation

To perform the RNAi experiment, double-stranded RNA (dsRNA) targeting the *Pv-Integrin β* gene was synthesized using the T7 in vitro transcription kit (Takara, Shiga, Japan) according to the method described by Yang et al. [32]. A total reaction volume of 20 μL was prepared by mixing the following components in sequence: 2 μL of DNA template, 2 μL of 10 × transcription buffer, 2 μL of A/U/C/GTP mixture (10 μM each), 0.5 μL of RNase inhibitor, and 2 μL of T7 RNA polymerase, with RNase-free water added to bring the total volume to 20 μL. The reaction mixture was incubated at 42 °C for 2 h, followed by the addition of 2 μL of RNase-free DNase I, after which it was mixed thoroughly and further incubated at 37 °C for 30 min. To ensure the quality of the dsRNA, phenol/chloroform extraction was performed, followed by ethanol precipitation. Before use, the working concentration of the dsRNA was adjusted to 3 μg/μL, and the samples were stored in an ultra-low-temperature (−80 °C) freezer. Following the same procedure, control dsGFP RNA was prepared, and the interference primer information is listed in Table 1.

### 4.6. Survival Rate Analysis After Pv-Integrin β Knockdown

Before analyzing the survival rate of shrimp after the knockdown of the target gene, we first verified the efficiency of the RNAi. Sixty shrimp were randomly divided into two groups, with thirty shrimp in each group (dsGFP + WSSV and dsIntegrin β + WSSV). The prepared dsRNA (approximately 30 μg) was injected into the abdominal segment of each shrimp using a microinjector. To enhance the RNAi efficiency, a second injection of dsRNA was performed 24 h later, followed by infection with WSSV (4.5 × 10^6^ copies). Total RNA was extracted from the hepatopancreas of the shrimp at 24, 36, and 48 hpi, and the expression level of *Pv-Integrin β* was detected using qRT-PCR, with 18 S rRNA as the internal reference gene. The quantitative primer information is listed in Table 1.

In the shrimp survival rate analysis experiment, 160 shrimp were randomly divided into four groups, with 40 shrimp in each group: PBS, WSSV-infected, dsGFP + WSSV, and dsIntegrin β + WSSV. After the knockdown of *Pv-Integrin β*, the survival rate of the shrimp was statistically analyzed. To ensure the robustness of the experiment, the above procedures were performed in triplicate. The experimental data were analyzed using GraphPad Prism 8.0 software (https://www.graphpad.com/, accessed on 8 December 2024), and statistical significance was assessed using a Kaplan–Meier plot (log-rank χ^2^ test).

### 4.7. Pathogen Detection After Pv-Integrin β Knockdown

To detect the replication of WSSV in *P. vannamei*, we selected muscle tissue for the quantification of pathogen copy numbers. After infecting the host, WSSV typically begins by invading the epidermis and subcutaneous tissues of the shrimp, causing white spots to appear on the body surface. As the infection progresses, the virus also invades the muscle tissue, leading to necrosis and softening of the muscles, which subsequently affects the shrimp’s motor function and survival rate. In this experiment, DNA was first extracted from the muscle tissues of the dsGFP + WSSV group and the dsIntegrin β + WSSV group at different time points (using the TIANamp Marine Animals DNA Kit, Tiangen Biotech Co., Ltd., Beijing, China). The pathogen detection experiment involved 60 shrimp, which were divided into 2 groups with 30 shrimp in each group. The WSSV copy number was then detected using TaqMan real-time PCR technology [33]. The specific primers and probes for WSSV are detailed in Table 1. The detection was performed using the THUNDERBIRD™ Probe qPCR Mix Kit (TOYOBO) and quantified with the Applied Biosystems^TM^ QuantStudio 1 real-time PCR system. The reaction mixture (20 μL) consisted of 10 μL of THUNDERBIRD Probe qPCR Mix, 0.6 μL each of F/R primers (0.2 μM), 0.4 μL of the WSSV probe (10 μmol/L), 0.1 μL of ROX reference dye, 2 μL of the DNA template, and 6.3 μL of nuclease-free distilled water. The PCR protocol included an initial denaturation process at 95 °C for 30 s, followed by 40 cycles of 95 °C for 5 s and 60 °C for 34 s. This experiment was conducted in three independent and parallel trials. Using this method, we were able to accurately detect the replication of WSSV in muscle tissue, providing reliable data for further research into the infection mechanisms of WSSV. Meanwhile, the relative expression levels of the WSSV encoded envelope protein gene *vp28* and the immediate-early gene (*IE1*) in the hepatopancreas were also detected by qRT-PCR after the knockdown of the target gene. The primer information is listed in Table 1.

### 4.8. Detection of Innate Immune Signaling Pathway Genes Following the Knockdown of the Pv-Integrin β Gene

To further investigate the changes in disease resistance pathway genes following the knockdown of the integrin receptor gene, qRT-PCR was employed to analyze the innate immune system. The experimental design was as follows: the shrimp were first subjected to a second injection of dsRNA (30 μg), followed by infection with WSSV (4.5 × 10^6^ copies). The relative expression levels of the genes were analyzed at 0, 2, 6, 12, 24, 36, and 48 hpi. A total of 90 shrimp were used in the experiment and divided into two groups: the experimental group (dsIntegrin β + WSSV) and the control group (dsGFP + WSSV). Hepatopancreas tissue was collected from the shrimp, and total RNA was extracted and reverse transcribed into cDNA for the detection of the mRNA expression levels of key genes; the genes analyzed included those related to the Toll, IMD, and STAT pathways. The primer information used for qRT-PCR is listed in Table 1. The experimental data were analyzed using the 2^−ΔΔCT^ method, and the experiment was independently repeated three times.

## 5. Conclusions

**Figure 9:**
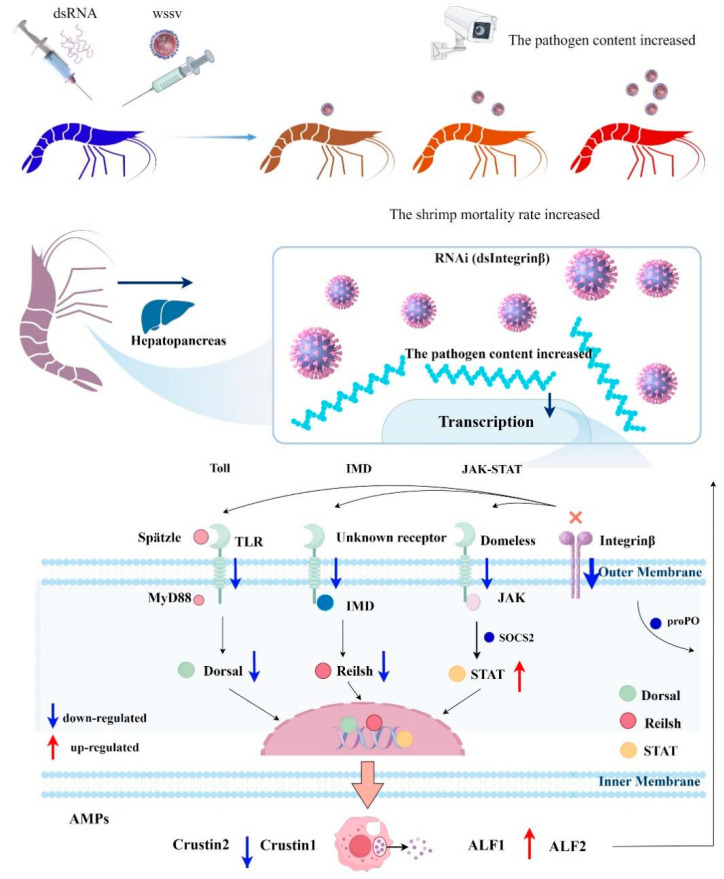
This figure is a schematic diagram illustrating the immune pathway of *P. vannamei* in response to certain diseases regulated by Integrin β. After the knockdown of *Integrin β*, it was observed that viral replication increased, and the survival rate of the shrimp decreased. Further exploration of its molecular mechanism revealed that Integrin β might affect other receptors and, through regulating the Toll, IMD, and STAT signaling pathways, regulate the expression of antimicrobial peptides, thereby influencing the innate immune system of *P. vannamei* against diseases. This schematic diagram was drawn using the Figdraw 2.0 software (https://www.figdraw.com/static/index.html#/, accessed on 10 May 2025). Explanation: The cross mark in the pattern diagram represents knockdown.

Through our research, we have proposed a molecular theoretical model where Integrin β regulates the antiviral innate immune system in the hepatopancreas of *P. vannamei* by influencing the expression of antimicrobial peptides. In fact, WSSV induces the expression of *Pv-Integrin β*. Knocking down *Pv-Integrin β* significantly increases the viral copy number of WSSV and also raises the cumulative mortality rate of the shrimp. Further analysis of the gene regulation mechanism reveals that the *Pv*-Integrin β protein may influence the expression of *Spätzle*, *Toll-like receptors*, and *Domeless*, thereby regulating the Toll, IMD, and STAT signaling pathways. This, in turn, modulates the expression of *ALF1*, *ALF2*, *Crustin1*, *Crustin2*, and *proPO* in the hepatopancreas, playing a crucial role in regulating the antiviral innate immune system in the hepatopancreas. The *SOCS2* gene, as a negative regulatory factor of STAT, may be involved in regulating the upregulation of *STAT* expression, thereby regulating the upregulation of *ALF1* and *ALF2* in order to participate in the antiviral immune response of shrimp. *Dorsal* and *Relish* regulate the downregulation of *Crustin1* and *Crustin2* expression (Figure 9).

## Figures and Tables

**Table 1:** The sequences of primers and probes used in this study.

Primer Name	Sequence (5′~3′)
**Amplified sequence**	
*Pv*-Integrin-β-F	GTCAGGGGTCTCTGCAAATCA
*Pv*-Integrin-β-R	CAGGTCTGTTCATCAGGTACT
**For the qRT-PCR assay**	
Integrin-β-F	TGCAAGAAGCCAGCAGATCC
Integrin-β-R	GAACATGTTGGCCCACTCCA
18 S-F	TATACGCTAGTGGAGCTGGAA
18 S-R	GGGGAGGTAGTGACGAAAAAT
VP 28-F	TTCTTTCACTCTTTCGGTCGT
VP 28-R	GCCAACTTCATCCTCATCAAT
IE 1-F	TGGCACAACAACAGACCCTA
IE 1-R	CTTTCCTTGAAGTACGAGAC
SOCS 2-F	CTGCCAAACGCCCACTTC
SOCS 2-R	CGTGGCAGGCATTGTGTG
MyD88-F	GGCAAAGGGCTATTGGAACTAT
MyD88-R	ATGATCCAGACACCTCTCGTATTC
proPO-F	TACATGCACCAGCAAATTATCG
proPO-R	AGTTTGGGGAAGTAGCCGTC
ALF1-F	TTACTTCAATGGCAGGATGTGG
ALF1-R	GTCCTCCGTGATGAGATTACTCTG
ALF2-F	GGCCATTGCGAACAAACTCAC
ALF2-R	GTCCATCCTGGGCACCACAT
Crustin1-F	GTAGGTGTTGGTGGTGGTTTC
Crustin1-R	CTCGCAGCAGTAGGCTTGAC
Crustin2-F	GGTACGTCTGCTGCAAGCC
Crustin2-R	CTGAGAACCTGCCACGATGG
Domeless-F	TCAGACAGGAGGTCTCATAC
Domeless-R	GTACCAGTGTGAAGCCTTAC
JAK-F	TACCCTGGTCTACGCTATAC
JAK-R	TGAGACGGTAGTACCCATTC
Spätzle-F	TGGGGACTCTCCTTACGATG
Spätzle-R	GGGAACAGACAGGTCTCCAA
Dorsal-F	GATGGAATGATAGAATGGGAAGC
Dorsal-R	CACTGGTACTCTTGTCTGGTGGTC
Relish-F	CTACATTCTGCCCTTGACTCTGG
Relish-R	GGCTGGCAAGTCGTTCTCG
IMD-F	CGGCTCTGCGGTTCACAT
IMD-R	CCTCGACCTTGTCTCGTTCCT
STAT-F	AGCCCCTGTCTGAGCGAAA
STAT-R	GGTGTTCTCTTGTGACCTTCATCA
TLR-F	GGGGTACCATGGTGTCAGGACTGGAGTCAGGCAACC
TLR-R	TGCTCTAGATACACTTTTCGAGTTTGATTTAACAAG
**Pathogen detection primers**	
WSSV-F	TGGTCCCGTCCTCATCTCAG
WSSV-R	GCTGCCTTGCCGGAAATTA
WSSV-probe	AGCCATGAAGAATGCCGTCTATCACACA
**For dsRNA synthesis**	
dsGFP-Fi	GCGTAATACGACTCACTATAGGCATCTTCTTCAAGGACGACGG
dsGFP-Ri	GCGTAATACGACTCACTATAGGAGTTCACCTTGATGCCGTTCT
*Pv*-Integrin-β-Fi	GCGTAATACGACTCACTATAGGACGACCGCAAGGGCAAAT
*Pv*-Integrin-β-Ri	GCGTAATACGACTCACTATAGGGACGCAGGGCTTGTAGTCTGT

## Data Availability

The data presented in this study are available in the article.
